# Mitigation and animal response to water stress in small ruminants

**DOI:** 10.1093/af/vfad049

**Published:** 2023-10-13

**Authors:** Sara Pérez, Jorge Hugo Calvo, Carlos Calvete, Margalida Joy, Sandra Lobón

**Affiliations:** Departamento de Ciencia Animal, Centro de Investigación y Tecnología Agroalimentaria de Aragón (CITA), 50059 Zaragoza, España; Departamento de Ciencia Animal, Centro de Investigación y Tecnología Agroalimentaria de Aragón (CITA), 50059 Zaragoza, España; Instituto Agroalimentario de Aragón – IA2 (CITA-Universidad de Zaragoza); Fundación Agencia Aragonesa para la Investigación y el Desarrollo (ARAID), 50018 Zaragoza, España; Departamento de Ciencia Animal, Centro de Investigación y Tecnología Agroalimentaria de Aragón (CITA), 50059 Zaragoza, España; Instituto Agroalimentario de Aragón – IA2 (CITA-Universidad de Zaragoza); Departamento de Ciencia Animal, Centro de Investigación y Tecnología Agroalimentaria de Aragón (CITA), 50059 Zaragoza, España; Instituto Agroalimentario de Aragón – IA2 (CITA-Universidad de Zaragoza); Departamento de Ciencia Animal, Centro de Investigación y Tecnología Agroalimentaria de Aragón (CITA), 50059 Zaragoza, España; Instituto Agroalimentario de Aragón – IA2 (CITA-Universidad de Zaragoza)

**Keywords:** adaptation, climate change, immune response, omics technologies, resilience

**Implications**

- Water stress is usually related to heat stress and decreased feed intake; so its effect is difficult to separate.
- Small ruminants are able to better adapt to water stress than other species, especially breeds from regions under harsh conditions.
- Mitigation strategies to water stress will be needed to maintain animal production.
- Breeding strategies are difficult to implement, and omics technologies are approaches that can help us to unravel the biological mechanisms involved in water stress adaptation.

## Introduction

**Figure 1.**
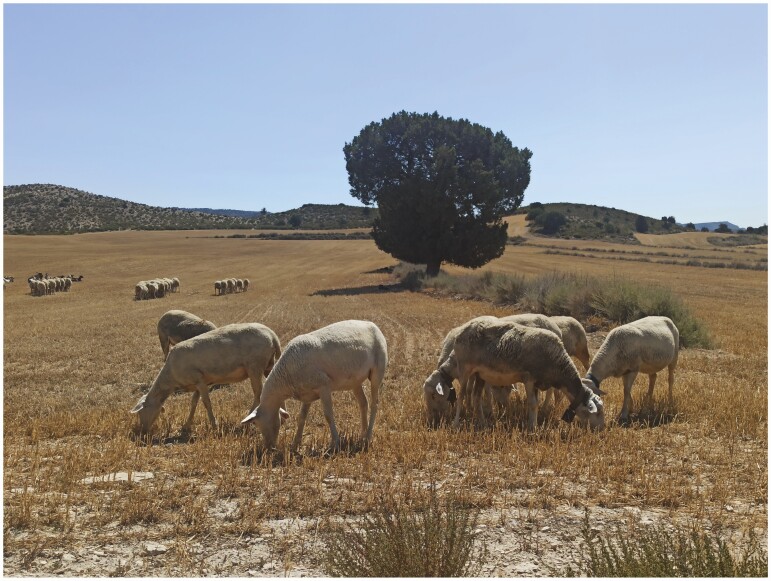
Sheep Flock of Rasa Aragonesa breed in “Los Monegros”, a dry region of Aragón, Spain.

Global climate change is a threat and a major challenge for livestock production because increasing temperatures and reduced water availability decrease forage quantity and quality. The effects of these climate changes are predicted to continue in forthcoming years, which highlight all the problems that water scarcity poses for small ruminant productivity, and for changes in animal physiological, immunological, and ethological aspects. Therefore, the future availability of animal products (e.g., milk, meat, fiber) will also be affected. Small ruminant management based on extensive or semi-extensive systems very much depends on environmental factors like precipitation and climatology. Moreover, water restriction or rationing policies can affect dairy or meat intensive small ruminant production. However, it is important to note that small ruminants can better tolerate water stress than large ruminants or monogastric species as the rumen has been reported to act as a water reservoir in well-adapted sheep (Silanikove, 1994). This water stress tolerance is closely related to breed, and adaptive and indigenous breeds from dry regions are more tolerant than selected breeds. Figure 1 shows a sheep flock of Rasa Aragonesa, a local breed, grazing stubbles in “Los Monegros” a dry region of Aragón (Spain).

The water requirement of livestock depends on different factors, such as species, diet, size, activity, environmental temperature, and physiological status. Therefore, it is difficult to indicate a specific amount for each species. In a recent trial carried out at CITA Research Center, the average daily water intake of 40 empty Rasa Aragonesa ewes (60 kg of BW) was 2.18 l. These ewes were allocated in individual pens and fed with a total mixed ration under an average temperature of 22 ºC and relative humidity of 56%. The environmental temperature has an important effect on the water intake, in this line Li et al. (2000) observed that ewes drank twice more water under warm (24.8 ºC) than cold (0.4 ºC) temperatures. Under heat stress, crossbreed goats drunk on average 7.4 l (Kaliber et al., 2009) whereas this amount was higher, 15.3 l, in lactating Aardi goats (Alamer, 2009). Therefore, the temperature and lactation status increase the water requirement. It is worth noting that cattle need higher amounts than small ruminants, steers on average drunk 37 liters daily (Ahlberg et al., 2019).

Water stress has been less studied in small ruminants than heat stress, which has been extensively investigated, and studies on mitigation and adaptation strategies to water stress are lacking.

Within this framework, this paper aims to highlight the main responses and effects of water stress on small ruminants, and the possible strategies that small ruminants can use to mitigate or adapt to water stress.

### Productive traits

Water stress affects productive traits mainly due to reduced feed intake, which helps to decrease water loss related with feed digestion and metabolism. Additionally, ruminants are able to cumulate water in the forestomach to use as a reserve to face water shortages. Simultaneous to reduced intake, rumen digestive capacity is enhanced by increasing crude protein digestibility (Hussein et al., 2020), but lower dietary energy intake has been recorded. Thus, animals present fat mobilization characterized by increased concentrations of cholesterol and free fatty acids (FFA) in blood, which has been observed in sheep (Jaber et al., 2004) and goats (Kaliber et al., 2016).

**Figure 2.**
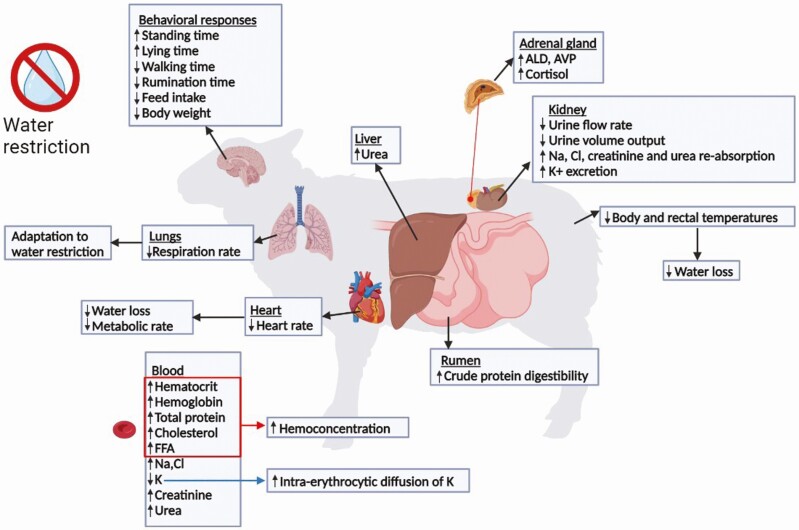
Physiological changes in sheep and goats under water restriction.

Evaporation is an important water loss process. It is directly related to metabolic rate and, therefore, the animal metabolic rate is decreased (i.e., heart rate, respiration rate, body temperature) to reduce water loss and to overcome decreased feed intake. Apart from the above-mentioned changes, animals acquire some thermoregulatory behavioral response by not only increasing duration of standing and lying, but also decreasing duration of walking and rumination time, as mechanisms to reduce consumed metabolic energy (Kaliber et al., 2016). These main effects are presented in Figure 2.

When animals suffer mild water restriction, besides the above mentioned adaptive responses, production traits can also be affected, such as reproduction efficiency, growth, and quality of milk and meat.

**Figure 3.**
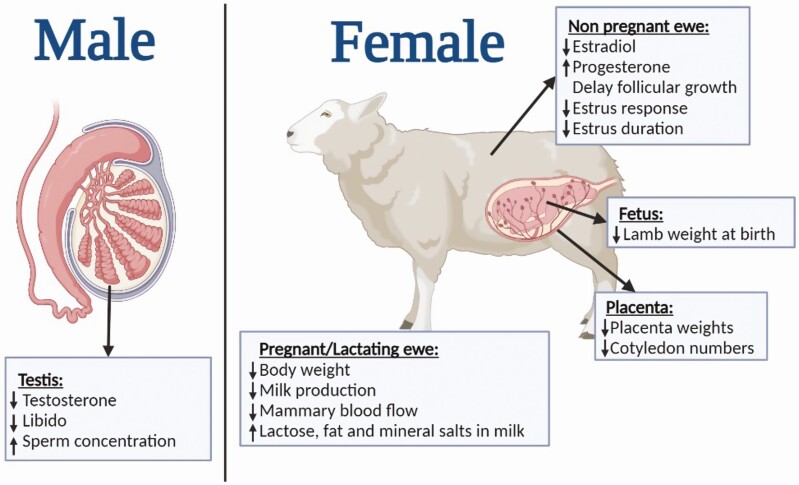
Effects on reproductivity traits in sheep under water restriction conditions.

Regarding reproduction, the effect of water restriction on male reproductive traits has been studied by Khnissi et al. (2016) with the Barbarine breed. Those authors showed that the rams which received water only every 4 d had reduced libido, decreased testosterone concentrations and a higher sperm concentration compared with the control rams, but no effects on semen traits were noted. In females, delayed follicular growth, a decrease in estrus response, estrus duration and estradiol in plasma, and increased progesterone have been reported (Naqvi et al., 2017). These outcomes are usually associated with reduced feed intake, which means that it is not simple to separate both effects of reduced water and feed intake on these traits. It is worth noting that some breeds are well-adapted to water deprivation during pregnancy and water deprivation does not alter lambing. Yetisgin and Sen (2020) worked with Awassi ewes and revealed that 50% water restriction reduces body weight (BW), placental weight, and number of cotyledons in pregnant ewes, and birth weights in lambs, but parturition in ewes is successful. This was possible thanks to placental and cotyledon efficiency, which augmented in restricted ewes by the ability to maintain pregnancy in water shortage circumstances. The main effects on reproductivity traits due water restriction are presented in Figure 3.

Researchers have paid considerable attention to the effect of water shortage during critical production periods, such as growing and lactating periods, including nutrition status, weight gain, meat quality, and milk secretion decreases with water restriction. Severe water shortage reduces BW upon slaughter, and hot and cold carcass yields, which can affect physio-chemical characteristics, mineral content and the fatty acid profile of meat, whereas dehydration and stress can darken meat color and decrease tenderness (Dos Santos et al., 2019; Akinmoladun et al., 2020; Araujo et al., 2022). Nevertheless, moderate water intake restrictions (up to 33% to 40% of voluntary intake or for 24 h) seem tolerated by Nadji or Santa Inês sheep breeds without compromising their production performance. This scenario suggests the possibility of implementing watering conservation strategies during water scarcity periods or as a resource-conservation measure for desert environments without endangering meat production in some breeds (Dos Santos et al., 2019; Araujo et al., 2022).

Regarding milk production, water shortage is usually associated with decreased milk yields. This reduction is because of slower mammary blood flow due to dehydration (Alamer, 2009). Reduced milk production in sheep and goats is usually accompanied by an increased in lactose, fat, and mineral salts concentration in milk (Casamassima et al., 2018), although decreased lactose concentrations have been reported in water-restricted sheep due to lactose secretion inhibition induced by cortisol (D’Ambrosio et al., 2018). As other production traits, milk production very much depends on resilience of the breed to water shortage. It has been shown that Aardi goats are very capable of withstanding water restrictions by economizing body water and striking new water balances with a 50% of water restriction, this adaptive response would allow dams to maximize lactation performance under water (and heat) stress conditions (Alamer, 2009). Conversely, lack of drinking water can significantly affect milk production levels in high production breeds that are poorly-adapted to water stress, like Lacaune dairy sheep, with tolerance to approximate water restrictions of up to 20% (Casamassima et al., 2018).

### Changes in blood parameters and hematology

Water economy entails major physiological changes in ruminants. When normal physiological limits are exceeded by water restriction, hormonal dynamics are altered to minimize water loss and to maintain osmotic balances in the internal milieu (Jaber et al., 2004). Stress can modify certain blood parameters, such as blood osmolarity, blood cell concentration, or hormone concentration. With plasma osmolality, an increase in sodium (Na^+^; Jaber et al., 2004; Kaliber et al., 2016) and chloride (Cl^−^) concentrations (Jaber et al., 2004), and a decline in potassium (K^+^) concentrations have been observed in sheep and goats with water restrictions, which cause blood hyperosmolality. Na^+^ and Cl^−^ (the predominant cation and anion) are the primary factors that determine extracellular fluid (ECF) osmolality in blood and are implicated in acid-base metabolism. To maintain an Na^+^ and Cl^−^ balance in body fluids, different small ruminant breeds increase renal retention due to the influence of aldosterone and arginine-vasopressin (Jaber et al., 2013). These two hormones are implicated in the control of the body fluid balance with water restriction, including renal excretion (Hamadeh et al., 2006; Ghanem et al., 2008), which decreases urine flow and volume (Jaber et al., 2013). Jaber et al. (2004) and Kaliber et al. (2016) report increased creatinine and urea in blood when sheep and goats were affected by water restriction, which can be partially a consequence of the re-absorption of these compounds, plus a reduced urine flow rate, and can also be due to the endogenous nitrogen source, which is related to proteolysis as a response to the protein/nitrogen intake deficiency observed when small ruminants are deprived of water. Potassium excretion through urine increases given its negative correlation with Na^+^, and blood K^+^ decreases, which are related to its intra-erythrocytic diffusion (Jaber et al., 2004)

Regarding hematology variables, water stress causes hemoglobin and hematocrit increases, along with a greater total protein concentration, which are related to a smaller plasma volume (hemoconcentration). Increased total protein could result from an increase in blood albumin and globulin, which are responsible for maintaining an osmotic equilibrium between the blood and tissue fluids required when feed intake declines (Khnissi et al., 2016).

### Immune response

Like other stressful conditions, it is assumed that water restriction can alter the immune function in small ruminants, mainly through the mediation of glucocorticoid stress hormones. Accordingly, endocrine stress markers, such as increased concentrations of cortisol or changes in differential leukocyte counts, have been reported in goats and sheep deprived of water (D’Ambrosio et al., 2018; Akinmoladun et al., 2020). However, changes in stress markers are not always associated with an impaired immunity function, and unchanged (Jaber et al., 2004; Ghanem et al., 2008) or even less concentrations of cortisol have been observed in water-restricted Awassi ewes (Hamadeh et al., 2006). Therefore, water stress effects on immunity should be assessed by examining specific immune parameters.

Ghassemi Nejad et al. (2014) studied water restriction effects after feeding on the immunoglobulin G concentration in already heat-stressed Corriedale ewes to find no difference between the control and water-restricted ewes. Conversely, Barbour et al., (2005) reported that water stress in Awassi ewes reduced immune response efficacy by reducing antibodies postvaccination. The noted different patterns of humoral response suggest that the immune response of water-deprived ewes was determined by the nature of not only immunogens, but also by physiological status of the individual because immunosuppression was more significant in lactating ewes than in unproductive water-deprived ewes. Finally, D’Ambrosio et al. (2018) used haptoglobin as a marker of systemic inflammation in sheep exposed to progressive decreasing water availability. They reported that water deprivation was related to a general inflammatory condition, which indicates that water stress activates an inflammatory pathway that includes haptoglobin, platelet factor 4 (PF4 cytokine), and ApoA-II lipoprotein with anti-oxidant and anti-inflammatory activity. The authors suggested that these markers might be combined to identify dehydrated animals and to monitor homeostasis restoral.

### Nutritional strategies to mitigate water stress

Apart from the physiology response to water stress conditions, some feeding strategies are adopted to mitigate water stress. One of the main effects of water restriction is reduced feed intake because water and feed intake are closely related. Therefore, diet should be of high quality to ensure the greatest possible nutrient intake. Van Der Walt et al. (1999) reported a similar intake in Mutton Merino wethers fed with low or medium protein diet with 50% water restriction. The wethers fed with medium protein presented slightly greater gains. Similarly, desert goats subjected to 40% water restriction lost more BW with low-quality hay than the goats fed with good-quality hay, and no differences in dry matter intake were reported (Ahmed and El Kheir, 2004). These studies highlight that a high-quality diet can help to alleviate water stress.

In addition, it is known that some supplements can also help to cushion the water stress impact. Although one of the most studied supplements is vitamin C, its supplementation is not a common practice in ruminants because they can biosynthesize under normal conditions. The interest in vitamin C supplementation is for its decrease in animals under stressful conditions (Akinmoladun et al., 2020) and its anti-oxidant capacity (free radical scavenging), which can improve the immune response (Chedid et al., 2014). For this reason, vitamin C administration has been studied in different stressed animals (i.e., pigs, broilers, goats, sheep, rabbits) with positive results (Chedid et al., 2014). In line with this, Awassi ewes supplemented with 2.5 g vitamin C/d under severe water restriction conditions presented with less BW loss and showed less marked increases in serum protein, globulin, and albumin concentrations compared with un-supplemented ewes, and without affecting FFA, cholesterol, glucose, serum cortisol and K^+^ (Ghanen et al., 2008.) Similarly, Jaber et al. (2011) studied vitamin C supplementation at different doses (3 g or 5 g daily, or two large doses of 10 g (8 d apart)) in Awassi ewes with a 3-d water restriction. They reported less BW loss compared with their counterparts without vitamin C, and no effect on fat mobilization was observed. Recently, Akinmoladun et al. (2020) concluded that vitamin C supplementation in Xhosa goats (an indigenous breed) under water stress and high temperature conditions cushioned impacts on loss BW, dry matter intake depression, and blood concentrations. Although vitamin C supplementation may be a good strategy to mitigate the effect of water stress on small ruminants by especially reducing loss BW, it is important to note that vitamin C was administered orally in the above-mentioned research, which could make flock management difficult or impossible, especially in extensive systems without available water. Therefore, it is necessary to continue searching for different supplements to mitigate adverse water stress effects and some that are easier to apply in different systems.

In general, nutritional strategies apart from vitamin C supplementation to alleviate water stress have been scarcely studied in ruminants. In contrast, heat stress and the ways to mitigate its effects have been extensively studied, especially in dairy animals. One way to mitigate heat stress is supplement dietary anti-oxidants to support immune function and oxidative status. Along these lines, selenium and vitamin E supplementation in heat-stressed sheep diminished adverse effects (Alhidary et al., 2015), with a down-regulation of inflammatory genes and an up-regulation of reactive oxygen species (Chauhan et al., 2014). As previously mentioned, water deprivation was related to a general inflammatory condition, and this strategy can be applied to mitigate the effects of this stress. Another successfully studied nutritional strategy was to increase fat in the diet with reductions in starch and fiber because fat is related to decreased metabolic heat. In addition, the inclusion of polyunsaturated fatty acids (PUFAs) in diet has been studied in recent decades and enhanced immune function in heat stress has been reported in sheep (Caroprese et al., 2012). Hence the field for further research is large, especially with a view to look for the best nutritional strategies that mitigate water stress effects on small ruminants. It would be interesting to study the inclusion of different anti-oxidants and PUFAs in diet to help ewes to balance the negative effect of water stress.

### The genetic component of water deprivation tolerance

The genetic control of this trait has barely been studied, and very few studies have been performed to determine how efficient ruminants are in using water. One of the first studies to measure efficiency in water use of ruminants was conducted by Ahlberg et al. (2019) in beef cattle. In that study, the water intake, residual water intake and water to gain ratio phenotypes were calculated, as were those related to feed intake, residual feed intake and dry matter intake, as well as growth traits like average daily gain. Residual water intake can be considered a water use efficiency indicator and, similarly to residual feed intake, it indicates the difference between the observed water intake of the animal and expected water intakes. These authors found moderate heritability for these traits to vary between 0.37 (for residual water intake) and 0.39 (for water intake and water to gain ratio), which indicates that their selection can be efficiently performed. The low genetic correlations between water intake and average daily gain (0.05) were also outstanding, but the genetic correlations with dry matter intake were moderate and positive (0.34), and residual water intake correlated negatively with residual feed intake (−0.57). Another study performed in beef cattle found similar results using corrected estimated values for these phenotypes to find positive Pearson correlations for water intake with dry matter intake (0.54; Pires et al., 2022). In small ruminants, only one study by Barros de Freitas et al. (2021) has estimated residual water intake and water intake in lambs to correlate these traits with food utilization efficiencies. These authors found that residual water intake correlated negatively with dry matter intake in relation to BW (−0.33) and the feed conversion rate (−0.44), but positively with the water to gain ratio (0.44). In summary, the more efficient animals in water use obtained high dry matter intake in relation to BW and better feed conversion rates. These water use efficiency-related results suggest the possibility of utilizing these traits in ruminant breeding schemes. However, more studies with these traits in small ruminants should be conducted before contemplating them in animal breeding. Furthermore, we have to consider that the estimation of the water intake phenotypes requires taking measurements on individual animals for traits like average daily gain, BW, or dry matter intake. This also implies either housing animals in individual pens or using automated recording systems that allow individual intakes to be measured.

Additionally, detecting efficient water use of the animal differs from detecting tolerant animals to water stress. The latter can tolerate water shortage by surviving up to 1 wk with little or no water, and by balancing animal adaptation and productivity, but generally with productive, reproductive, and immunity impaired (Barbour et al., 2005). However, the literature that has studied the genetic basis of how small ruminants tolerate water shortage is scarce. Indeed only genome studies that compare breeds to detect the genomic regions linked with local adaptation to harsh environmental conditions have been performed (Yang et al., 2016; Bertolini et al., 2018; Manuza et al., 2023). In these studies, the genomic adaptation regions include those related to the ability to adapt to harsh environmental conditions, and include not only water scarcity, but also others like hot temperatures or poor-quality forage. In this context, the candidate genes related to water retention and re-absorption in renal cells and blood vessels in kidneys have been identified when comparing Chinese native sheep breeds to a breed adapted to desert conditions using whole genome sequencing (Yang et al., 2016). These genes were involved in the arachidonic acid metabolism, the renin-angiotensin system and oxytocin signaling pathways. Another study, performed with four goat breeds (Angora, Boer, Nubian, Saanen) genotyped with the SNP chip 50 K to identify selection footprints as a response of environmental adaptation, revealed a few markers potentially under selection for Boer and Saanen (Manuza et al., 2023). In the Boer breed, the *SLC4A4* gene previously identified in the study by Yang et al. (2016) in sheep was identified in one significant run of a homozygosity island. This protein is overexpressed in the basolateral membrane of renal proximal convoluted tubules, and plays a critical role in the bicarbonate and vasopressin-regulated water re-absorption pathways. Finally, a landscape genomic study using Illumina CaprineSNP50 BeadChip in 144 goat breeds showed multiple signatures of selection to be associated with bioclimatic environmental variables (Bertolini et al., 2018). Bioclimatic variables were grouped based on, for example, temperature and precipitation, differences, and can, thus, indirectly indicate water availability, but without separating them from other confounding factors.

**Figure 4.**
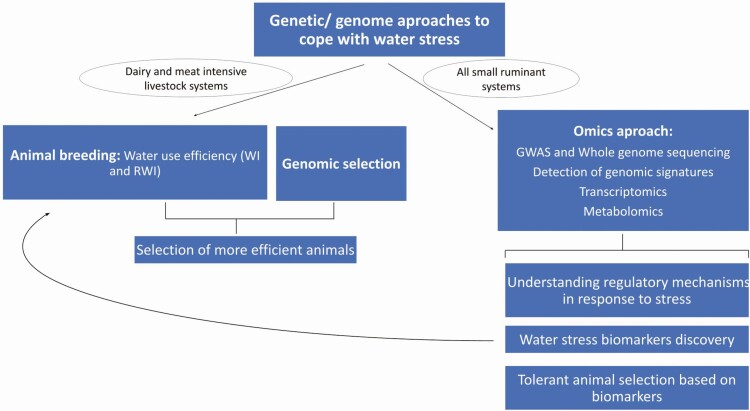
Genetic and genomic approaches to manage water stress depending on the ruminant production system. WI: Water intake; RWI: Residual water intake; GWAS: genome-wide association studies.

Breeding strategies are difficult to implement in extensive systems given the need to house animals to take accurate measurements of the phenotype in meat small ruminants. However in dairy or meat intensive small ruminant production, efficient water use measurements can be implemented into a breeding scheme, including genomics selection because of its moderate heritability. More research is necessary using “omics” technologies to explore water shortage of the animal tolerance capacity, especially in arid and water-limiting areas. This is why it is necessary to develop population-specific models with an accurate measurement of extreme values for the water stress phenotype to identify the genes that underlie water shortage tolerance, and to then acquire knowledge about the regulation mechanisms response to these traits. Apart from detecting genomic signatures studies, genome-wide association studies (GWAS) and transcriptome analyses can help to identify the key genes or pathways involved in the water stress tolerance trait. Metabolomic studies can also provide the metabolites and metabolic pathways associated with water stress to be used as biomarkers for the selection of water deprivation tolerance animals. These biomarkers can be employed as an indirect measure of stress tolerance. Metabolomics, together with transcriptomics, can play a key role in searching for a network of connections that link stress and genes, metabolites, phenotypes, and biomarkers (Figure 4).

## Conclusion

The response to water stress in small ruminants has been studied mainly in well-adapted breeds to arid environments, which shows high resilience to water stress. The mechanism of adaptation and mitigation to this stress is very complex, with a positive relation to dehydration and notably reduced feed intake. In addition, heat and water stress are difficult to separate and jointly affect the aforementioned traits in a high percentage of situations. Thus, specific studies using animal models that are more adapted to water shortage should be performed. Moreover, as studies that have explored strategies to mitigate water stress are lacking, it would be interesting to test different supplements to alleviate the impacts on animal. The inclusion of anti-oxidant and PUFAs has been explored in heat stress to reduce negative impacts and could, therefore, be also studied under water stress conditions. However, as water scarcity is expected to be longer in the future, selecting more adapted animals from such conditions is necessary. Although breeding strategies are difficult to implement, using omics technologies could help us to understand the biological mechanisms involved in animals that are better adapted to water shortage, and to identify the water stress biomarkers that can be applied to select better adapted animals.

## About the Author(s)

**Sara Pérez** is a PhD student at the Department of Animal Science in the Agrifood of Research and Technology Centre (CITA) of Aragon (Spain). She studied for a Bachelor’s in Biology at the University Autónoma of Madrid (UAM), specializing in Cellular Biology and Genetics. Then she studied an MSc in Research in Veterinary Sciences at Universidad Complutense of Madrid (UCM) and other MSc in Bioinformatics and Computational Biology also in UAM. She is currently working on a project that studies the biomarkers associated with water restriction to find stress tolerance ewes, characterize candidate genes or genomic regions associated with this stress tolerance, and thus, identify genomic signatures of selection. Her PhD also studies the influence of stress tolerance and sainfoin supplementation under water restriction and parasite infection.

**Jorge Hugo Calvo, PhD** in Veterinary Science from Zaragoza University, is a senior ARAID researcher at the Agrifood of Research and Technology Centre (CITA), Zaragoza, Spain. His research is focused on the application of genomic tools in animal science, mainly in ruminants, including animal breeding, nutrigenomics, the genetic background of the reproductive traits, and also in animal health. He is also interested in the study of the tolerance genetic basis to different biotic and abiotic stresses in ruminants in semi-arid bioclimatic regions.

**Carlos Calvete** has a university degree and a PhD in Veterinary Sciences from the University of Zaragoza (Spain) and, currently, he is a researcher at the Animal Science Department in the Agri-Food Research Center of Aragón (CITA, Spain). His main research interests concern epidemiology and control of parasitic diseases in small ruminants, especially the improvement of anthelmintic use in sheep.

**Margalida Joy** is a Researcher of Animal Science at the Agrifood of Research and Technology Centre of Aragón (CITA), Aragon, Spain. She received the PhD from the University Autonoma of Barcelona. She did her postdoctoral training at the University of California, Davis. Her research is focused on ruminant production systems, with a holistic approach integrating the relationships between breeds, nutrition, management, pasture use, feeding systems and animal products, within a socioeconomic and environmental sustainability context.

**Sandra Lobón** graduated in Veterinary Science from the University of Zaragoza. She developed her PhD in sheep systems at the Agrifood of Research and Technology Centre of Aragón (CITA, Spain), where she is currently working as a postdoctoral researcher. She has been working on projects focused on ruminants, evaluating efficiency and resilience, grazing and intensive systems, alternative feeding, and optimization of protein resources. Her professional goal as a researcher is to evaluate sustainable and climate change resilient systems improving the quality of the final product.
